# Serum α-klotho concentrations during preimplantation can predict aging or quality of human oocytes and clinical pregnancy rates

**DOI:** 10.1186/s40064-016-1706-7

**Published:** 2016-01-20

**Authors:** Takashi Takemura, Midori Okabe

**Affiliations:** Reproductive Medicine Institute Japan, Nakano-ku, Chuo, 3-37-12, Tokyo, 164-0011 Japan

**Keywords:** Serum α-klotho concentrations, Serum 25 (OH) D levels, Aging or quality of human oocytes

## Abstract

**Background:**

To discover simple biomarkers to evaluate the aging or quality of human oocytes and clinical pregnancy rates is needed. However, the association among serum α-klotho concentrations during preimplantation, the aging or quality of human oocytes and clinical pregnancy rates has not been investigated.

**Findings:**

The serum α-klotho concentrations during preimplantation decreased due to aging (p < 0.001), whereas the maturation rates of human oocytes (p < 0.001) and the fertilization rates (p < 0.001) improved in association with increased serum α-klotho concentrations. Furthermore, multiple logistic regression analysis showed that the clinical pregnancy rates were influenced by serum α-klotho concentrations during preimplantation (p < 0.001), the patient’s age (p = 0.003), maturation rates of human oocytes (p < 0.001), fertilization rates (p < 0.001) and the serum 25 (OH) D levels (p < 0.001) regardless of race (p = 0.29) and BMI (p = 0.96).

**Conclusion:**

The serum α-klotho concentrations during preimplantation would be a simple biomarker in order to predict the aging or quality of human oocytes and clinical pregnancy rates.

## Background

A simple biomarker to evaluate the aging or quality of human oocytes and clinical pregnancy rates is required. On the other hand, since klotho protein was detected in 1997 (Kuro-o et al. 2007), klotho is intensively researched. Klotho is an ageing-modulated protein expressed mainly in the kidneys and choroid plexus (Wolf et al. 2014). Furthermore, α-klotho is known as an anti-aging molecule. The α-klotho gene mutant mice have been shown to have short life-spans and multiple aging phenotypes analogous to those observed in humans, such as skin atrophy, osteoporosis, ectopic calcification, atherosclerosis, and pulmonary emphysema (Nakanishi et al. 2015). Moreover, increases in α-klotho concentrations in human serum positively promote human health (Yamazaki et al. 2010). For examples, α-klotho is known as cardio-renal protective protein (Lee et al. 2014). Serum α-klotho concentration may be novel and useful early markers of diabetic renal injury (Lee et al. 2014). Therefore, increases in α-klotho concentrations in human serum positively may improve the aging or quality of human oocytes and clinical pregnancy rates. However, an association between serum α-klotho concentrations during preimplantation and the aging or quality of human oocytes has not been investigated.

Hochbaum et al. (2011) previously identified *klo*-*2*, a homologue of α-klotho gene, as one candidate direct target of DAF-12 (a homologue of vitamin D receptor) in model organism *Caenorhabditiselegans* (Hochbaum et al. 2011). On the other hand, the association studies between serum α-klotho concentrations and vitamin D were conducted (Koyama et al. 2015; Shardell et al. 2015). However, an association between serum α-klotho concentrations during preimplantation and vitamin D in infertile patients has not been investigated.

## Methods

### Study population

Healthy non-pregnant women participated in the present study (Table 1). Written informed consent was obtained from subjects prior to enrollment. From March 2012 to April 2015, a total of 633 women were enrolled.

**Table 1:** Demographic and clinical characteristics of the patients

Characteristics	Value
No. patients	633
Mean maternal age (range) (years)	36.84 (30–45)
Race—no. (%)	
Asian	443 (70 %)
White	178 (28.1 %)
Black	12 (1.9 %)
Current smoker—no. (%)	0 (0 %)
Intracytoplasmic sperm injection (ICSI) (%)	633 (100 %)
Mean serum alpha-klotho concentrations (pg/mL) (range)	593.14 (123.77–1227.1)
Mean maturation rate (range) (%)	39.8 (0–100)
Mean fertilization rate (range) (%)	37.9 (0–100)
Pregnancy rate (%)	33
Mean body mass index (BMI)	21.4 (16.9–31.6)
Mean serum 25 (OH) D levels (nmol/L) (range)	74.8 (30–144)

### Measurement of serum α-klotho levels

Blood samples were drawn from a forearm vein in the morning after overnight fasting. Sera were obtained by centrifugation and immediately stored at −30 °C. Serum α-klotho concentrations were evaluated during preimplantation using the human soluble α-klotho Assay Kit (TAKARA BIO Inc., Japan).

### Measurement of serum 25 (OH) D levels and pregnancy

Serum 25 (OH) D levels were measured using enzymeimmunoassay (Immunodiagnostic Systems Inc., Fountain Hills, AZ, USA). Furthermore, pregnancies were based on detection of a gestational sac (GS).

### The ovarian stimulation protocol

Eligible patients in the present study were treated with the ovarian stimulation regimen (mild stimulation protocol). In the mild stimulation protocol, patients received clomiphene citrate at 100 mg/day from day 3 to day 7 followed by human chorionic gonadotropin (hMG) injections 1–5 times. 0.25 mg of gonadotropin-releasing hormone (GnRH) antagonist was started every 24 h when the leading follicle diameter reached 14 mm. With transvaginal ultrasound monitoring, when the diameter of a dominant follicle reached 18 mm, all the patients were administered with hCG (5000 IU) intramuscularly. Transvaginal sonographically guided oocyte retrieval was performed 36 h after hCG injection.

### The evaluation of oocyte maturation rates and the fertilization rates

In the samples of oocytes from the women, the presence of matured oocytes at metaphase II (MII) was confirmed. The oocytes with polar body were regarded as mature MII oocytes, and were used for normal intracytoplasmic sperm injection (ICSI). Furthermore, success rates of maturation and fertilization were determined under microscopic observation (Olympus IX71, Japan) by two embryologists at our institute. Moreover, maturation rates were assessed by the number of MII oocytes/total number of oocytes used for in vitro maturation at 24 or 48 h. Fertilization rates were assessed with the number of 2PN oocytes/total number of oocytes used for ICSI at 24 or 48 h after ICSI.

### Approval of institutional review board

We, authors, confirm that the work described has been carried out in accordance with The Code of Ethics of the World Medical Association (Declaration of Helsinki). Furthermore, all of the experiments were approved by the institutional review board at Reproductive Medicine Institute Japan.

### Statistical analyses

All of the statistical tests were performed using Dr. SPSS II for Windows (SPSS Japan, Inc., Tokyo), and significance was defined as p < 0.05 (two-tailed). Continuous values were expressed as the mean values ± standard deviation (SD). Statistical analyses were investigated by using single logistic regression analysis and multiple logistic regression analysis.

## Results

### Demographic and clinical characteristics of the patients

Demographic and clinical characteristics of the patients were shown as Table 1. Mean maternal age was 36.84 years (range 30–45). All patients were treated by ICSI as assisted reproductive technology. Ovarian stimulation protocol was one (see “Methods” section). Pregnancy rates was 33.0 %. Furthermore, all patients were non-smoker. Moreover, Asian was 70.0 % (n = 443), White was 28.1 % (n = 178) and Black was 1.90 % (n = 12).

### Associations among serum α-klotho concentrations during preimplantation, the fertilization rates and the maturation rates

**Fig. 1:**
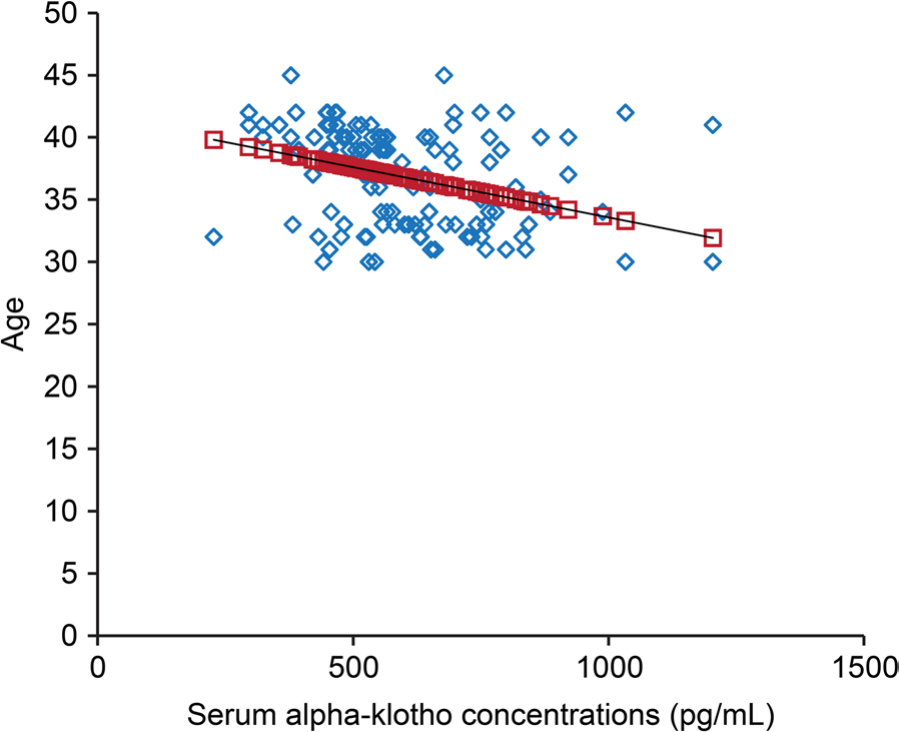
An association between serum α-klotho concentrations during preimplantation and age. X-axis: serum α-klotho concentrations during preimplantation (pg/mL), y-axis: age

**Fig. 2:**
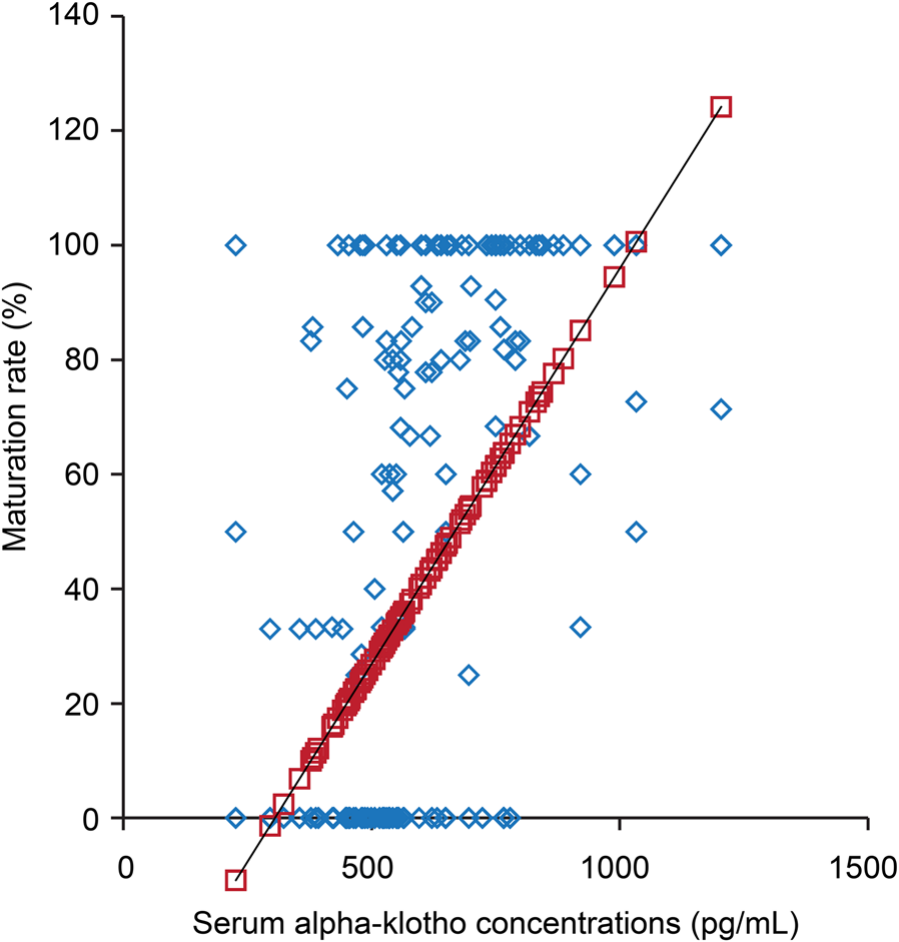
An association between serum α-klotho concentrations during preimplantation and maturation rates. X-axis: serum α-klotho concentrations during preimplantation (pg/mL), Y-axis: maturation rates (%)

**Fig. 3:**
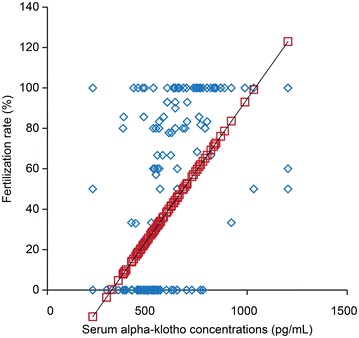
An association between serum α-klotho concentrations during preimplantation and fertilization rates. X-axis: serum α-klotho concentrations during preimplantation (pg/mL), Y-axis: fertilization rates (%)

The serum α-klotho concentrations during preimplantation were decreased by aging (Fig. 1, p < 0.001), while the maturation rates (Fig. 2, p < 0.001) and the fertilization rates (Fig. 3, p < 0.001) were improved by increasing of the serum α-klotho concentrations during preimplantation.

### Associations among serum α-klotho concentrations during preimplantation, the clinical parameters [BMI and serum 25 (OH) D levels]

**Fig. 4:**
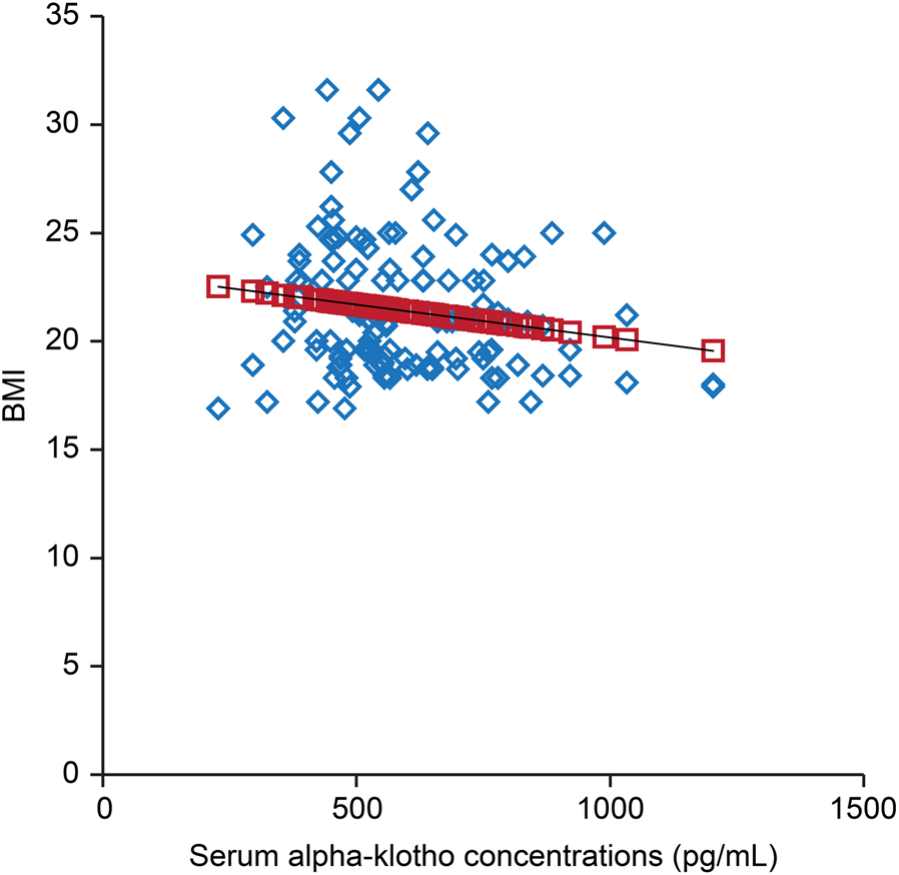
Associations between serum α-klotho concentrations during preimplantation and BMI. X-axis: serum α-klotho concentrations during preimplantation (pg/mL), Y-axis: BMI

**Fig. 5:**
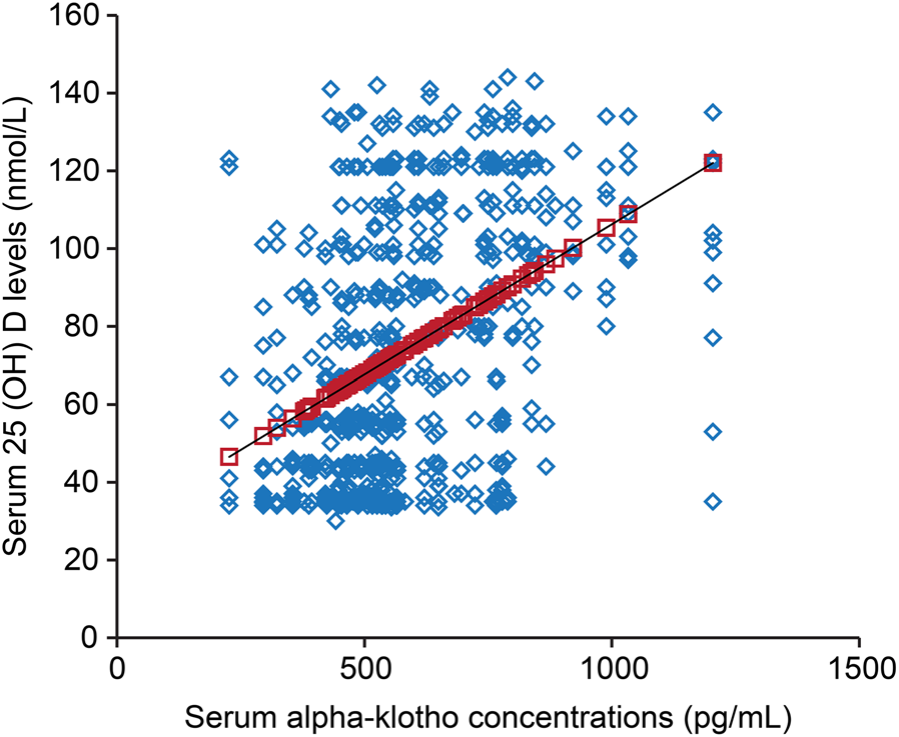
Associations between serum α-klotho concentrations during preimplantation and serum 25 (OH) D levels. X-axis: serum α-klotho concentrations during preimplantation (pg/mL), Y-axis: serum 25 (OH) D levels

BMI was negatively associated with the serum α-klotho concentrations during preimplantation (Fig. 4, p < 0.001). Furthermore, the serum 25 (OH) D levels were positively associated with the serum α-klotho concentrations during preimplantation (Fig. 5, p < 0.001).

### Multiple logistic regression analysis

Multiple logistic regression analysis (Table 2) showed that the clinical pregnancy rates were influenced by serum α-klotho concentrations during preimplantation (p < 0.001), the patient’s age (p = 0.003), maturation rates of human oocytes (p < 0.001), fertilization rates (p < 0.001) and the serum 25 (OH) D levels (p < 0.001) regardless of race (p = 0.29) and BMI (p = 0.96). After the multivariate analysis, the clinical pregnancy rates were positively associated with the patient’s age, serum α-klotho concentrations, maturation rates of human oocytes, fertilization rates and the serum 25 (OH) D levels.

**Table 2:** Multiple logistic regression analysis

	t value	p value
Age	−2.95616	0.003232
Serum alpha-klotho concentrations (pg/mL)	10.5659	<0.0001
Maturation rate (%)	7.68942	<0.0001
BMI	−0.04677	0.962714
Serum 25 (OH) D levels (nmol/L)	6.220646	<0.0001
Race	−1.06139	0.288925
Fertilization rate (%)	6.789421	<0.0001

### Cutoff for alpha-klotho concentrations during preimplantation that was correlating with clinical pregnancy rates

Mean serum alpha-klotho concentrations during preimplantation was 593.14 pg/mL in the present study. Furthermore, according to a previous report, 562 pg/mL in healthy adults (n = 142, mean) age 20 years old or over (Yamazaki et al. 2010). Therefore, when we considered 550 pg/mL a cutoff for alpha-klotho that was correlating with clinical pregnancy rates, with this cutoff value, 65.0 % sensitivity and 54.0 % specificity for prediction of pregnancy was achieved.

Furthermore, when we considered 600 pg/mL a cutoff for alpha-klotho that was correlating with clinical pregnancy rates, with this cutoff value, 81.0 % sensitivity and 65.0 % specificity for prediction of pregnancy was achieved.

Moreover, when we considered 700 pg/mL a cutoff for alpha-klotho that was correlating with clinical pregnancy rates, with this cutoff value, 70.0 % sensitivity and 50.0 % specificity for prediction of pregnancy was achieved.

Therefore, considering these factors, we considered 600 pg/mL a cutoff for alpha-klotho that was correlating with clinical pregnancy rates in the present study.

## Discussion

The data of a report implicate DNA double-strand break (DSB) repair efficiency as an important determinant of oocyte aging in women (Titus et al. 2013). However, more simple biomarker in order to evaluate the aging or quality of human oocytes and clinical pregnancy rates is needed in the clinical setting. Furthermore, while current understanding of the molecular biology of the α-klotho may offer new insights into its function and role in aging, the possibility of the serum α-klotho concentrations during preimplantation in order to evaluate the aging or quality of human oocytes and clinical pregnancy rates has not been investigated. In this regard, our present prospective study is the first study reporting that the serum α-klotho concentrations during preimplantation can predict the aging or quality of human oocytes and clinical pregnancy rates significantly. However, as pregnancy rate depend on many confounding factors, although we considered many confounding factors by multiple logistic regression analysis, further another factors should be considered.

On the other hand, the serum 25 (OH) D levels were positively associated with the serum α-klotho concentrations during preimplantation in the present study. Furthermore, the administration of 1,25-(OH)2D3 induced the expression of klotho in the kidney in mice (Tsujikawa et al. 2003). Therefore, the serum α-klotho concentrations during preimplantation may be improved by vitamin D supplementation. Further studies will be needed.

## Conclusion

In conclusion, the serum α-klotho concentrations during preimplantation would be useful as a biomarker in order to predict the aging or quality of human oocytes and clinical pregnancy rates.

## References

[CR1] Hochbaum D, Zhang Y, Stuckenholz C, Labhart P, Alexiadis V, Martin R, Knölker HJ, Fisher AL (2011). DAF-12 regulates a connected network of genes to ensure robust developmental decisions. PLoS Genet.

[CR2] Koyama D, Sato Y, Aizawa M, Maki T, Kurosawa M, Kuro-O M, Furukawa Y (2015). Soluble α Klotho as a candidate for the biomarker of aging. Biochem Biophys Res Commun.

[CR3] Kuro-o M, Matsumura Y, Aizawa H, Kawaguchi H, Suga T, Utsugi T, Ohyama Y, Kurabayashi M, Kaname T, Kume E, Iwasaki H, Iida A, Shiraki-Iida T, Nishikawa S, Nagai R, Nabeshima YI (2007). Mutation of the mouse klotho gene leads to a syndrome resembling ageing. Nature.

[CR4] Lee EY, Kim SS, Lee JS, Kim IJ, Song SH, Cha SK, Park KS, Kang JS, Chung CH (2014). Soluble α-klotho as a novel biomarker in the early stage of nephropathy in patients with type 2 diabetes. PLoS One.

[CR5] Nakanishi K, Nishida M, Harada M, Ohama T, Kawada N, Murakami M, Moriyama T, Yamauchi-Takihara K (2015). Klotho-related molecules upregulated by smoking habit in apparently healthy men: a cross-sectional study. Sci Rep.

[CR6] Shardell M, Semba RD, Kalyani RR, Hicks GE, Bandinelli S, Ferrucci L (2015). Serum 25-hydroxyvitamin D, plasma klotho, and lower-extremity physical performance among older adults: findings from the InCHIANTI study. J Gerontol A Biol Sci Med Sci.

[CR7] Titus S, Li F, Stobezki R, Akula K, Unsal E, Jeong K, Dickler M, Robson M, Moy F, Goswami S, Oktay K (2013). Impairment of BRCA1-related DNA double-strand break repair leads to ovarian aging in mice and humans. Sci Transl Med.

[CR8] Tsujikawa H, Kurotaki Y, Fujimori T, Fukuda K, Nabeshima Y (2003). Klotho, a gene related to a syndrome resembling human premature aging, functions in a negative regulatory circuit of vitamin D endocrine system. Mol Endocrinol.

[CR9] Wolf I, Shahmoon S, Ben Ami M, Levy-Shraga Y, Mazor-Aronovitch K, Pinhas-Hamiel O, Yeshayahu Y, Hemi R, Kanety H, Rubinek T, Modan-Moses D (2014). Association between decreased klotho blood levels and organic growth hormone deficiency in children with growth impairment. PLoS One.

[CR10] Yamazaki Y, Imura A, Urakawa I, Shimada T, Murakami J, Aono Y, Hasegawa H, Yamashita T, Nakatani K, Saito Y, Okamoto N, Kurumatani N, Namba N, Kitaoka T, Ozono K, Sakai T, Hataya H, Ichikawa S, Imel EA, Econs MJ, Nabeshima Y (2010). Establishment of sandwich ELISA for soluble alpha-Klotho measurement: age-dependent change of soluble alpha-Klotho levels in healthy subjects. Biochem Biophys Res Commun.

